# Sarcoma classification by DNA methylation profiling in clinical everyday life: the Charité experience

**DOI:** 10.1186/s13148-022-01365-w

**Published:** 2022-11-15

**Authors:** Siyer Roohani, Felix Ehret, Eilís Perez, David Capper, Armin Jarosch, Anne Flörcken, Sven Märdian, Daniel Zips, David Kaul

**Affiliations:** 1 Charité – Universitätsmedizin Berlin, corporate member of Freie Universität Berlin and Humboldt-Universität zu Berlin, Department of Radiation Oncology, Augustenburger Platz 1, 13353 Berlin, Germany; 2 Berlin Institute of Health at Charité – Universitätsmedizin Berlin, Charitéplatz 1, 10117 Berlin, Germany; 3 Charité - Universitätsmedizin Berlin, Berlin, German Cancer Consortium (DKTK), partner site Berlin, and German Cancer Research Center (DKFZ), 69120 Heidelberg, Germany; 4 Department of Neuropathology, Charité – Universitätsmedizin Berlin, corporate member of Freie Universität Berlin and Humboldt‑Universität zu Berlin, Charitéplatz 1, 10117 Berlin, Germany; 5 Institute of Pathology, Charité – Universitätsmedizin Berlin, corporate member of Freie Universität Berlin and Humboldt-Universität zu Berlin, Charitéplatz 1, 10117 Berlin, Germany; 6 Department of Hematology, Oncology and Tumor Immunology, Charité – Universitätsmedizin Berlin, corporate member of Freie Universität Berlin and Humboldt-Universität zu Berlin, Augustenburger Platz 1, 13353 Berlin, Germany; 7 Centre for Musculoskeletal Surgery, Charité – Universitätsmedizin Berlin, corporate member of Freie Universität Berlin and Humboldt-Universität zu Berlin, Augustenburger Platz 1, 13353 Berlin, Germany

**Keywords:** Sarcoma, Bone, Soft tissue, DNA methylation, Methylation profiling, Profiling, Classifier, Clinical experience

## Abstract

**Background:**

Sarcomas are a heterogeneous group of rare malignant tumors with more than 100 subtypes. Accurate diagnosis remains challenging due to a lack of characteristic molecular or histomorphological hallmarks. A DNA methylation-based tumor profiling classifier for sarcomas (known as sarcoma classifier) from the German Cancer Research Center (Deutsches Krebsforschungszentrum) is now employed in selected cases to guide tumor classification and treatment decisions at our institution. Data on the usage of the classifier in daily clinical routine are lacking.

**Methods:**

In this single-center experience, we describe the clinical course of five sarcoma cases undergoing thorough pathological and reference pathological examination as well as DNA methylation-based profiling and their impact on subsequent treatment decisions. We collected data on the clinical course, DNA methylation analysis, histopathology, radiological imaging, and next-generation sequencing.

**Results:**

Five clinical cases involving DNA methylation-based profiling in 2021 at our institution were included. All patients’ DNA methylation profiles were successfully matched to a methylation profile cluster of the sarcoma classifier’s dataset. In three patients, the classifier reassured diagnosis or aided in finding the correct diagnosis in light of contradictory data and differential diagnoses. In two patients with intracranial tumors, the classifier changed the diagnosis to a novel diagnostic tumor group.

**Conclusions:**

The sarcoma classifier is a valuable diagnostic tool that should be used after comprehensive clinical and histopathological evaluation. It may help to reassure the histopathological diagnosis or indicate the need for thorough reassessment in cases where it contradicts previous findings. However, certain limitations (non-classifiable cases, misclassifications, unclear degree of sample purity for analysis and others) currently preclude wide clinical application. The current sarcoma classifier is therefore not yet ready for a broad clinical routine. With further refinements, this promising tool may be implemented in daily clinical practice in selected cases.

## Introduction

Sarcomas are a heterogeneous group of rare malignant tumors with more than 100 subtypes listed in the current World Health Organization (WHO) classification [1]. Half of all sarcoma entities lack characteristic molecular and histomorphological hallmarks frequently leading to misclassification and discrepancies among pathologists [2–5].

A DNA methylation-based profiling classifier (sarcoma classifier) from the German Cancer Research Center (Deutsches Krebsforschungszentrum (DKFZ)) is now employed in selected cases at our institution as a valuable tool to guide tumor classification and subsequent treatment decision in challenging cases [5, 6]. Initially introduced as a successful tool for the classification of central nervous system (CNS) tumors, the classification system was extended to sarcomas [7, 8].

Data on the daily clinical experience with the sarcoma classifier are lacking. Herein, we describe the clinical course of five sarcoma cases undergoing thorough pathological and reference pathological examination as well as methylation-based profiling and their impact on subsequent treatment decisions.

## Materials and methods

Five cases with DNA methylation-based sarcoma classification between January and December 2021 were reviewed [6]. DNA methylation signals were processed using the R/Bioconductor package minfi (version 1.4.0.) as previously described [6, 9]. For visualization and dimensionality reduction, t-distributed stochastic neighbor embedding (t-SNE) was computed via the R package Rtsne (version 0.15) using the 25,000 most variable CpG sites according to standard deviation, 5000 iterations, and a perplexity value of 10 [10, 11].

Medical records were searched for clinical data on histopathological, immunohistochemical, and molecular pathological analyses from the institutional and reference pathology departments, medical tumor board (MTB) reports, imaging data, surgery reports, chemotherapy and radiotherapy treatment plans, past medical history, and outcome data.

## Results

### Patient characteristics

#### Patient 1

**Fig. 1:**
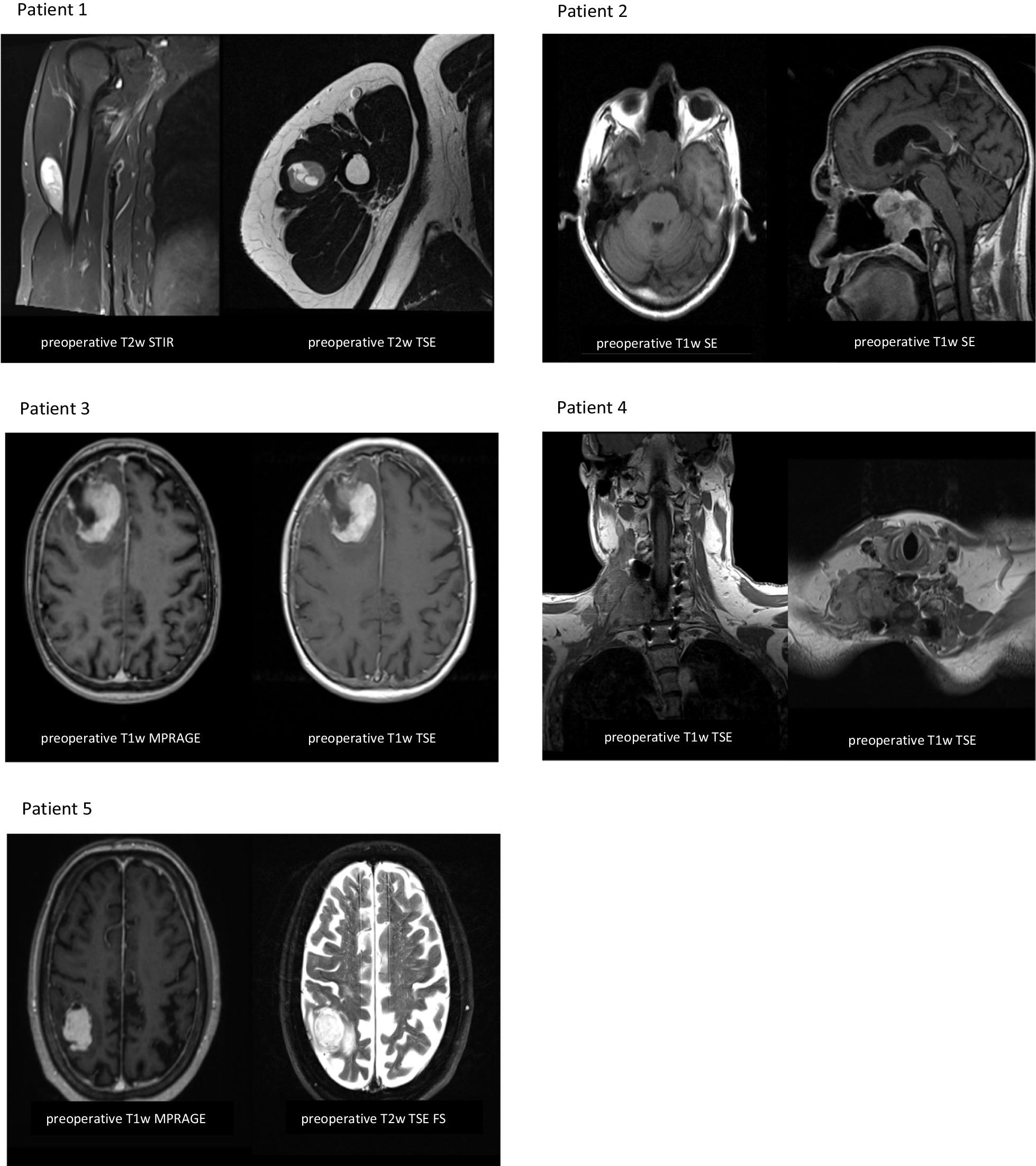
Preoperative MRI images. Representative MRI at the time of radiographic diagnosis and before treatment. *FS* fat saturation, *MPRAGE* magnetization prepared rapid gradient echo, *T1w* T1-weighted image, *T2w* T2-weighted image,* TSE* Turbo spin echo

**Fig. 2:**
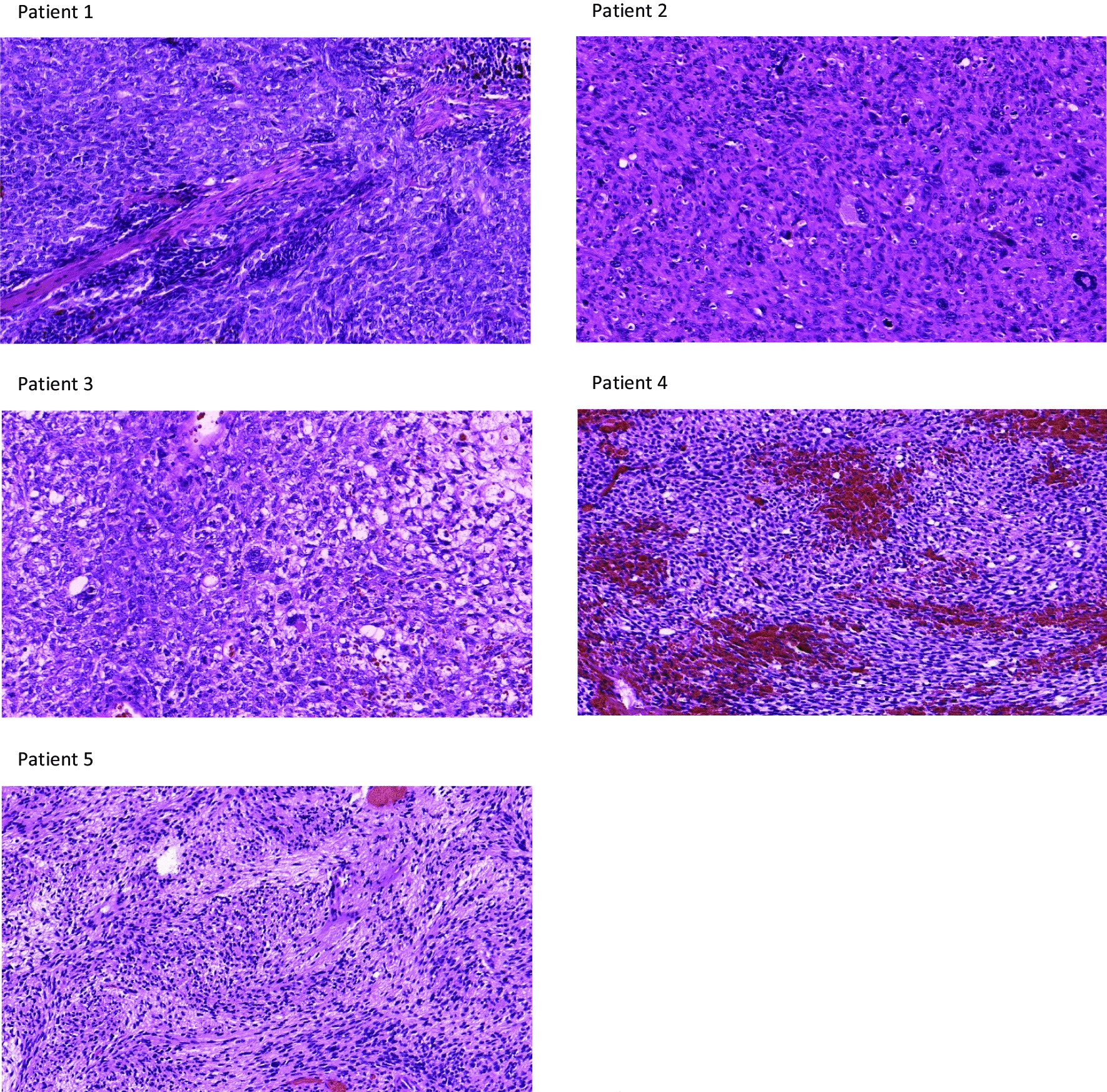
Hematoxylin–eosin (HE) staining, 200-fold enlargement. *Patient 1* Pleomorphic tumor with high cellular density and malignant spindle-shaped, sarcomatoid tumor tissue not clearly indicating one tumor entity. *Patient 2* Poorly differentiated, mesenchymal tumor tissue with storiform growth pattern diagnosed as a UPS. *Patient 3* Very poorly differentiated malignant cells with high proliferative activity lacking further characteristics that indicate one specific tumor entity. *Patient 4* Pleomorphic, mesenchymal tumor tissue with high cellular density suggestive of a previously diagnosed synovial sarcoma. *Patient 5* Highly proliferative, pleomorphic and partially spindle-shaped tumor tissue not clearly indicating one diagnosis

**Fig. 3:**
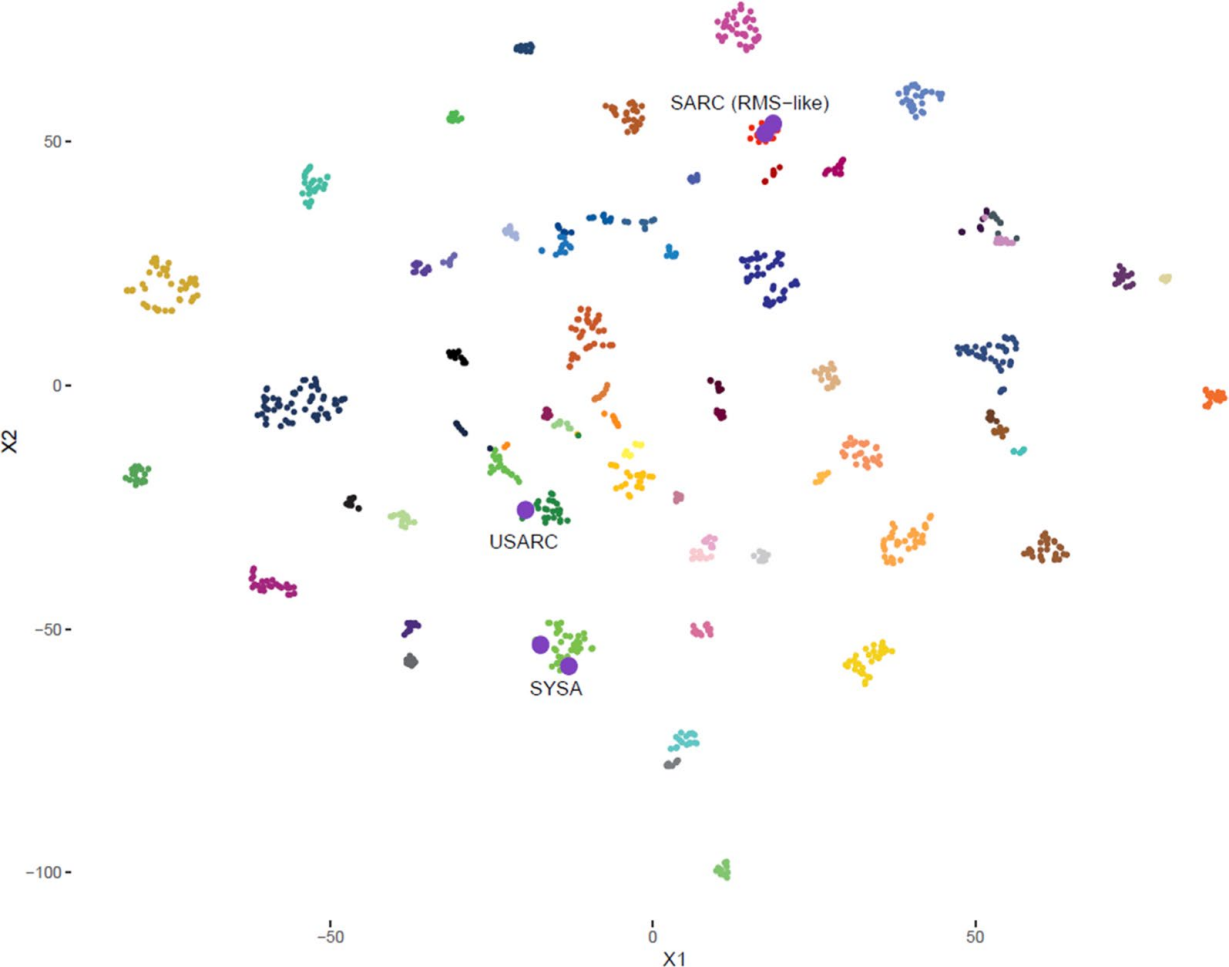
T-SNE analysis of DNA methylation data of all five cases presented in this series (highlighted by larger size and purple color) together with the sarcoma classifier reference cohort of 62 tumor methylation classes and three non-neoplastic control methylation classes. Full details of each methylation class can be found in the original publication [6]. Reference methylation class abbreviations: SARC (RMS-like), methylation class sarcoma (RMS-like) (17 cases); USARC, methylation class undifferentiated sarcoma (26 cases); SYSA methylation class synovial sarcoma (39 cases)

In October 2021, a 38-year-old male presented to the neurosurgical outpatient clinic with a 10-month history of pain and tenderness without neurological deficits in the right shoulder. Magnetic resonance imaging (MRI) showed a progressive, solid-appearing, round lesion in the right deltoid muscle compartment measuring 5.6 × 2.3 × 1.6 cm in size and minor areas suggestive of necrosis (Fig. 1). No bone infiltration or lymphadenopathy was visible. The radiologist suspected a benign schwannoma or myxoid liposarcoma. After resection, an initial neuropathological examination revealed a malignant spindle-shaped, sarcomatoid tumor (Fig. 2). Differential diagnoses after imaging and conventional histology included malignant peripheral nerve sheath tumor (MPNST) or synovial sarcoma. DNA methylation analysis detected a profile matching synovial sarcoma (calibrated score: 0.99, Fig. 3). No copy number variations were identified. Molecular examination conducted by the reference pathologist revealed a translocation on chromosome 18 (SS18/SYT) suggestive of synovial sarcoma. After metastatic spread was ruled out by computed tomography (CT), the MTB recommended second surgery with wide resection, since the initial resection was performed with close margins. A wide resection was then conducted, after which the patient received radiotherapy with 2 Gy daily to a total dose of 50 Gy followed by a boost of 2 Gy daily up to 16 Gy. On the day of the last follow-up in April 2022, no radiological evidence of disease was present and the patient was in good condition.

#### Patient 2

In July 2021, a 65-year-old male with recurrent epistaxis was referred to an otorhinolaryngology outpatient clinic for endoscopy. A biopsy from the nostrils revealed a high-grade undifferentiated pleomorphic sarcoma (UPS). The pathology department at our hospital reexamined the specimen and confirmed the diagnosis (Fig. 2). The patient had a history of an undifferentiated squamous cell carcinoma (SCC) with parts of a neuroendocrine differentiated carcinoma of the nose, paranasal sinuses, and the frontobasal cranium treated with surgery (R1 resection) and postoperative radiotherapy in 1999. To reaffirm the diagnosis, particularly in light of the prior tumor entity and the possibility of a radiation-induced angiosarcoma, DNA methylation analysis was performed. The classifier suggested a UPS (calibrated score 0.91, Fig. 3). The tumor was unfavorably located in the epipharynx (5.1 × 4.8 cm) with infiltration of the skull base (Fig. 1). Upon MTB recommendation, transsphenoidal resection with no postoperative radiographic signs of tumor residuals was conducted. Hyperfractionated postoperative radiotherapy was halted after 11 fractions when an interim CT scan showed progressive disease. The patient was referred to palliative systemic therapy with adriamycin and ifosfamide. He developed disease progression despite five cycles of chemotherapy six months later. MTB recommended a chemotherapy switch to trabectedin. Two weeks later the patient was admitted to the emergency room with progressive somnolence and severe local progress. The patient passed away soon thereafter.

#### Patient 3

In December 2020, an 80-year-old female patient with new-onset personality changes and transient aphasia was transferred to the emergency department from an external hospital after a cranial MRI had revealed a 4 × 3.7 cm mass lesion in the right frontal lobe (Fig. 1). Prompt surgical resection was conducted. The medical history of the patient included a well-differentiated grade II oligometastatic neuroendocrine tumor (NET) of the pancreas with lymph node and liver metastases, first diagnosed in 1996. Consequently, CNS metastases of the NET were considered a differential diagnosis. Apart from very poorly differentiated malignant cells with high proliferative activity, no further characteristics pointed toward a certain tumor entity (Fig. 2). Particularly, there were no clear indications for neuroendocrine active cells in the specimen. While the brain tumor classifier did not match a certain methylome pattern, the sarcoma classifier revealed a methylation profile compatible with a malignant Rhabdomyosarcoma-like (RMS-like) tumor with DICER1 mutation (calibrated score: 0.99, Fig. 3) [8, 12]. In line with this, p53 staining showed high expression and accumulation in the cell nucleus. Reference pathological examination added positive MyoD1 and myogenin staining. Based on histomorphology and immunohistochemistry, the reference pathologist initially suspected a UPS. However, the classifier matched to RMS-like tumors with DICER1 mutation and the subsequent next-generation sequencing (NGS) confirming DICER1 mutation established the diagnosis. A missense mutation of one Alpha Thalassemia/Mental Retardation Syndrome X-Linked allele and a splice site mutation with varying allele frequency were also detected; however, there was no immunohistochemical loss of ATRX expression.

Because of the limited life expectancy due to metastatic NET, the MTB opted for postoperative radiotherapy. After ruling out further tumor spread by CT (except for the known NET in the abdomen), the patient started radiotherapy but passed away one week later in an outside hospital.

#### Patient 4

In August 2021, a follow-up MRI in a 44-year-old male patient with known synovial sarcoma with an SS18/SYT translocation revealed the fourth paravertebral local recurrence at the level of the third cervical vertebra (Fig. 1). The patient had already undergone multiple resections and postoperative radiotherapies without complications. Another surgery was performed, and a neuropathological examination confirmed the diagnosis of synovial sarcoma (Fig. 2). Due to the young age and the multiple recurrences, the MTB initiated molecular diagnostics including NGS to recruit the patient for studies on targeted therapies in the translational Molecularly Aided Stratification for Tumor Eradication program of the National Center for Tumor Diseases (MASTER) [13]. However, NGS brought forth mutations atypical for synovial sarcomas, including cAMP-dependent protein kinase type I-alpha regulatory subunit (PRKAR1A) on chromosome 17q24.2 and a likely pathogenic mutation of the neurofibromin 2 (NF2) gene on chromosome 22q. On the other hand, known mutations of synovial sarcomas (e.g., TP53, phosphatase and tensin homolog (PTEN), Catenin Beta-1 (CTNNB1), and Adenomatosis polyposis coli (APC)) were not detected. In light of the unusual molecular finding, further clarification was required using DNA methylation profiling. Formalin-fixed and paraffin-embedded (FFPE) material from prior resections was analyzed with the sarcoma classifier and unequivocally confirmed the diagnosis of synovial sarcoma (calibrated score: 0.99, Fig. 3). However, molecular testing in the MASTER program did not reveal specific cellular or molecular targets of therapy. The patient was therefore treated with gemcitabine and docetaxel with palliative intention. Follow-up imaging four months later revealed local recurrence, pulmonary spread, and progressive spinal infiltration.

#### Patient 5

A 79-year-old male was admitted to the emergency department with a first-time event of a secondary generalized seizure in January 2021. Cranial MRI revealed a 3.2 × 2.3 × 3 cm mass located in the right-sided parietal lobe (Fig. 1) with microhemorrhage around the borders and surrounding edema. Due to a history of localized prostate carcinoma, brain metastasis was suspected. After subtotal resection with intraoperative radiotherapy (30 Gy), initial neuropathological examination of the specimen showed a pleomorphic, proliferative, and partially spindle-shaped tumor (Fig. 2). Apart from vimentin, the tumor did not show any characteristic surface markers in the immunohistochemical examination, including prostate-specific markers and ATRX. The tumor did show nuclear accumulation of p53 making the diagnosis challenging, although the initial suspicion of prostate cancer metastasis was highly unlikely at this point. The brain tumor classifier (Version v11b4) was unable to assign the methylation profile to a CNS tumor entity [8]. Subsequent methylation analysis for sarcomas matched the tumor to a malignant spindle cell sarcoma with RMS-like features which commonly carry a DICER1-mutation (calibrated score: 0.99, Fig. 3) [12]. Reference pathological examination additionally found expression of Myogenin and MyoD1 on immunohistochemistry and confirmed a rhabdomyogenic phenotype. Shortly thereafter, NGS showed a hotspot missense mutation on one DICER1 allele and a heterozygous loss of the other wild type allele leading to a biallelic inactivation of the gene. Moreover, a frameshift mutation of TP53 and a nonsense mutation of ATRX were found, matching the initial immunohistochemical findings of p53 accumulation and loss of ATRX expression. After the diagnosis of a primary intracranial RMS-like sarcoma was established and confirmed by the reference pathology, the MTB recommended a whole-body positron emission tomography scan with ^18^F-fluorodeoxyglucose showing no signs of extracranial disease. Eight months later, a local recurrence was treated with surgery, achieving gross total resection. Neuropathological examination confirmed the previous diagnosis. MTB recommended postoperative radiotherapy; however, the patient did not adhere to this recommendation. Two months thereafter, cranial MRI again revealed a local recurrence. The MTB recommendation for radiotherapy or palliative chemotherapy were both declined by the patient. In January 2022, the patient was admitted to the neurological intensive care unit with a generalized seizure despite anticonvulsant medication. In a shared decision-making process with the patient’s family, the decision in favor of best supportive care was made. The patient was transferred to a hospice and passed away one year after the initial diagnosis.

## Discussion

Over the past years, DNA methylation profiling has gained a role in the characterization of sarcomas with inconclusive morphological and molecular features and helped to find novel subgroups [14–20]. The development of a DNA methylation-based classifier for sarcomas enables the analysis of patient samples to improve diagnostics, especially in challenging cases [6].

In the first external validation study, Lyskjær and De Noon et al. analyzed 820 sarcoma samples (external validation cohort) [7]. The study brought forth important findings, particularly when compared to the DKFZ’s validation cohort (*n* = 428) [6]: The classifier’s rate of matches was higher in the DKFZ’s validation cohort (75% vs 61% in the external validation cohort). However, among the cases that received a match in the DKFZ cohort (i.e., calibrated score ≥ 0.9), the rate of findings concordant with the histological/molecular diagnosis was lower compared to the external validation cohort (61% + 7% discrepant cases with evidence in favor of the classifier’s diagnosis in the DKFZ cohort vs 88% in the external validation cohort). Moreover, the misclassification rate of 1% in the DKFZ’s cohort was favorably low compared to the substantially higher rate of 12% misclassifications in the external validation study. Importantly, the classifier challenged and changed the histological diagnosis after re-evaluation in 7% (29/428) of the DKFZ cohort, while this only occurred in 0.7% (6/820) in the external validation cohort.

The underlying reasons for these striking differences remain unclear. Possible explanations are the greater, institute-specific experience of the DKFZ with the preparation and handling of tumor samples that improves the matching rate. In support of this idea, the DKFZ brain tumor classifier also showed higher matching rates in the own validation cohort compared to external validation studies [8, 21, 22]. Moreover, the reference database of the sarcoma classifier as an open-access platform may have grown as new methylation datasets were uploaded, possibly explaining the higher rates of concordant diagnoses in the external validation study, although it does not explain the higher misclassification rates. Furthermore, the smaller sample size in the DKFZ validation cohort (*n* = 428 samples vs. *n* = 820 in the external study) may also account for the differences. Additionally, the authors’ suspicion that the DKFZ reference dataset is more homogeneous in terms of variety for each sarcoma subtype compared to the samples of the external validation study is possibly explaining the higher rates of misclassification [7]. Another explanation may be institutional differences in histopathological reassessment when the classifiers’ diagnosis challenges the histopathological examination [5].

Herein, we describe the first clinical experience with the sarcoma classifier: In three patients, the classifier confirmed the given diagnosis or aided in finding the correct diagnosis in light of contradictory data and differential diagnoses. In two patients with intracranial tumors, the classifier changed the clinical suspicion of metastatic disease to a novel diagnostic tumor group (RMS-like sarcomas with DICER1 mutation). In patient 1, the diagnosis of synovial sarcoma would have likely been established without the classifier, since, according to histomorphology, synovial sarcoma or MPNST was initially suspected. Nevertheless, the classifier substantiated initial suspicion before reference pathological examination and gave the important hint towards synovial sarcoma which was unequivocally confirmed. In patient 4, the classifier functioned as a tool to reconfirm the diagnosis in light of ambiguous molecular findings.

A notable advantage of the classifier in this situation is the fact that demands on the samples for the analysis are relatively low: Common FFPE samples obtained from the patient one year earlier were retrieved and analyzed by the classifier without difficulties. This is especially useful in patients with long oncological disease courses when the initial diagnosis is challenged and reassessed retrospectively as it frequently occurred with CNS tumors since the introduction of the brain tumor classifier [23].

An important requirement for sample preparation is the level of purity [6]. Interestingly, the external validation study found increasing sample purity to indeed correlate with higher rates of matches [7]. However, there was no difference in sample purity between tumors that were classified correctly (matching their histological diagnosis) and tumors that were classified incorrectly (contradicting the molecular and histologic diagnosis) [7]. Importantly, sample purity was similar in both cohorts [6, 7]. Nevertheless, it is a logical assumption that a certain level of sample purity is necessary for analysis; one possible solution to improve accuracy would be to subtract known methylation patterns from contaminating cells (e.g., lymphocytes), as suggested by the authors [6].

The previous medical history of patient 2 (undifferentiated SCC with parts of a neuroendocrine differentiated carcinoma, status post radiotherapy) together with the highly undifferentiated tumor specimen imposed a diagnostic challenge. The classifier aided by confirming the UPS diagnosis thereby ruling out radiation-induced angiosarcoma. Interestingly, recent methylation profiling data revealed new, distinct methylation clusters for angiosarcomas induced by radiation with a possible diagnostic value that may also be implemented in the classifier’s database [19]. The intracranially located RMS-like tumors with DICER1 mutations found in patients 3 and 5 represent novel sarcoma entities not previously described by the WHO classification of bone and soft tissue tumors [1, 6, 12]. The classifier’s role was critical and substantially changed the management in both patients. While metastatic disease of a known NET was ruled out in patient 3, the classifier’s results also established a completely new diagnosis in patient 5. Both cases confirm previous findings on intracranial RMS-like tumors with DICER1 mutation typically carrying genetic characteristics unequivocally detectable by DNA methylation analysis and NGS while the histomorphology is highly diverse and tumor cells appear heterogeneous [12]. No broad clinical experience exists on this new tumor entity. However, the authors who initially described the entity in a series of 22 almost exclusively intracranially located tumor samples suspected aggressive clinical courses based on histomorphological and genetic features (brisk mitotic activity, undifferentiated heterogeneous tissue, TP53 and MAP-kinase pathway mutations in the majority of cases, etc.) [12]. Due to the distinct clinical and molecular presentation, the tumors have been added to the WHO classification of CNS tumors [12, 24].

The classifier’s ability to unbiasedly decipher and group entirely new tumor entities based on epigenetic methylation patterns has undoubtedly revolutionized tumor diagnostics as seen by the impact of the brain tumor classifier since its initial introduction [5, 8, 24–28]. From our clinical perspective and the cases described herein, the sarcoma classifier functioned as a valuable ancillary diagnostic tool. However, in its current version, the sarcoma classifier is still a research tool facing multiple challenges before it can be applied in daily clinical routine: most importantly, the high rate of non-classifiable cases (25% in the DKFZ cohort; 39% in the external validation cohort); misclassifications of 12% in the external validation cohort and at least 1% in the DKFZ cohort; matches of samples not included in the reference data set (*n* = 42 of 163 in the external validation cohort); the unanswered question on the minimum degree of sample purity for stable classification precision, high costs and other limitations [5–7].

In the clinical setting, we therefore consider the classifier’s result as a second unbiased diagnostic finding. When the classifier matches and is concordant with a diagnosis suspected by other examinations, the diagnosis can most likely be accepted. If the classifier matches and contradicts the previous findings, a thorough reassessment and additional molecular diagnostics indicated by the classifier should follow as recently recommended by Koelsche et al. [5]. Further studies are required to evaluate the diagnostic value of different calibrated score ranges below 0.9 (e.g., 0.5–0.9 vs. < 0.5), all of which are currently defined as “no matches”.

In the future, the inclusion of new sarcoma subtypes not currently represented in the classifier as well as dataset expansion will most likely increase the classifier’s precision [5]. More interestingly, the combination of methylation analysis with evolving multiomics characterization of sarcomas will synergistically tackle the diagnostic ambiguity of sarcomas by improving individual tumor profiling [5, 29, 30]. Our data presented herein are obviously limited by the small number of selected cases where successful matching contributed to the clinical process. Further clinical experiences and validation studies for the sarcoma classifier are therefore awaited with great interest.

## Data Availability

Data are available upon request from the authors.

## Abbreviations

APC: Adenomatosis polyposis coli
ATRX: Alpha Thalassemia/Mental Retardation Syndrome X-Linked
CTNNB1: Catenin Beta-1
CNS: Central nervous system
CT: Computed tomography
DKFZ: Deutsches Krebsforschungszentrum (German Cancer Research Center)
FFPE: Formalin-fixed and paraffin-embedded
FS: Fat saturation
HE: Hematoxylin–eosin
MPNST: Malignant peripheral nerve sheath tumor
MPRAGE: Magnetization prepared rapid gradient echo
MRI: Magnetic resonance imaging
MTB: Medical tumor board
NGS: Next-generation sequencing
NET: Neuroendocrine tumor
PRKAR1A: Protein kinase type I-alpha regulatory subunit
PTEN: Phosphatase and tensin homolog
SS18/SYT: Synovial sarcoma translocation, chromosome 18
RMS-like: Rhabdomyosarcoma-like
SARC (RMS-like): Rhabdomyosarcoma-like, abbreviation in t-SNE analysis
SCC: Squamous cell carcinoma
SYSA: Synovial sarcoma, abbreviation in t-SNE analysis
T1w: T1-weighted image
T2w: T2-weighted image
t-SNE: T-distributed stochastic neighbor embedding
TSE: Turbo spin echo
UPS: Undifferentiated pleomorphic sarcoma
USARC: Undifferentiated sarcoma, abbreviation in t-SNE analysis
WHO: World Health Organization

## References

[1] Soft Tissue and Bone Tumours, WHO Classification of Tumours, 5th Edition, Volume 3, 2020.

[2] Pasquali S, Bonvalot S, Tzanis D, Casali PG, Trama A, Gronchi A (2019). Treatment challenges in and outside a network setting: soft tissue sarcomas. Eur J Surg Oncol.

[3] Loong HH, Blay JY, Munhoz RR (2019). International collaborations and regional challenges in providing specialist multidisciplinary sarcoma care. Am Soc Clin Oncol Educ Book.

[4] Ray-Coquard I, Montesco MC, Coindre JM, Dei Tos AP, Lurkin A, Ranchère-Vince D (2012). Sarcoma: concordance between initial diagnosis and centralized expert review in a population-based study within three European regions. Ann Oncol.

[5] Koelsche C, von Deimling A. Methylation classifiers: Brain tumors, sarcomas, and what's next. Genes Chromosomes Cancer. 2022. 10.1002/gcc.23041 35388566

[6] Koelsche C, Schrimpf D, Stichel D, Sill M, Sahm F, Reuss DE (2021). Sarcoma classification by DNA methylation profiling. Nat Commun.

[7] Lyskjaer I, De Noon S, Tirabosco R, Rocha AM, Lindsay D, Amary F (2021). DNA methylation-based profiling of bone and soft tissue tumours: a validation study of the 'DKFZ Sarcoma Classifier'. J Pathol Clin Res.

[8] Capper D, Jones DTW, Sill M, Hovestadt V, Schrimpf D, Sturm D (2018). DNA methylation-based classification of central nervous system tumours. Nature.

[9] Aryee MJ, Jaffe AE, Corrada-Bravo H, Ladd-Acosta C, Feinberg AP, Hansen KD (2014). Minfi: a flexible and comprehensive Bioconductor package for the analysis of Infinium DNA methylation microarrays. Bioinformatics.

[10] van der Maaten L, Hinton G (2008). Visualizing data using t-SNE. J Mach Learn Res.

[11] JH K. Rtsne: T-distributed stochastic neighbor embedding using a barnes-hut implementation 2015 [Available from: https://cran.r-project.org/web/packages/Rtsne/Rtsne.pdf.

[12] Koelsche C, Mynarek M, Schrimpf D, Bertero L, Serrano J, Sahm F (2018). Primary intracranial spindle cell sarcoma with rhabdomyosarcoma-like features share a highly distinct methylation profile and DICER1 mutations. Acta Neuropathol.

[13] (DKFZ) DK. NCT (National Center for Tumor Diseases) DKTK (German Cancer Consortium) MASTER (Molecularly Aided Stratification for Tumor Eradication) 2022 [Available from: https://www.nct-heidelberg.de/forschung/molecular-stratification/master.html.

[14] Koelsche C, Hartmann W, Schrimpf D, Stichel D, Jabar S, Ranft A (2018). Array-based DNA-methylation profiling in sarcomas with small blue round cell histology provides valuable diagnostic information. Mod Pathol.

[15] Koelsche C, Stichel D, Griewank KG, Schrimpf D, Reuss DE, Bewerunge-Hudler M (2019). Genome-wide methylation profiling and copy number analysis in atypical fibroxanthomas and pleomorphic dermal sarcomas indicate a similar molecular phenotype. Clin Sarcoma Res.

[16] Seki M, Nishimura R, Yoshida K, Shimamura T, Shiraishi Y, Sato Y (2015). Integrated genetic and epigenetic analysis defines novel molecular subgroups in rhabdomyosarcoma. Nat Commun.

[17] Röhrich M, Koelsche C, Schrimpf D, Capper D, Sahm F, Kratz A (2016). Methylation-based classification of benign and malignant peripheral nerve sheath tumors. Acta Neuropathol.

[18] Wu SP, Cooper BT, Bu F, Bowman CJ, Killian JK, Serrano J, et al. DNA methylation-based classifier for accurate molecular diagnosis of bone sarcomas. JCO Precis Oncol. 2017;2017. 10.1200/PO.17.00031 PMC5772901 29354796

[19] Weidema ME, van de Geer E, Koelsche C, Desar IME, Kemmeren P, Hillebrandt-Roeffen MHS (2020). DNA methylation profiling identifies distinct clusters in angiosarcomas. Clin Cancer Res.

[20] Kommoss FKF, Stichel D, Schrimpf D, Kriegsmann M, Tessier-Cloutier B, Talhouk A (2020). DNA methylation-based profiling of uterine neoplasms: a novel tool to improve gynecologic cancer diagnostics. J Cancer Res Clin Oncol.

[21] Priesterbach-Ackley LP, Boldt HB, Petersen JK, Bervoets N, Scheie D, Ulhøi BP (2020). Brain tumour diagnostics using a DNA methylation-based classifier as a diagnostic support tool. Neuropathol Appl Neurobiol.

[22] Pickles JC, Fairchild AR, Stone TJ, Brownlee L, Merve A, Yasin SA (2020). DNA methylation-based profiling for paediatric CNS tumour diagnosis and treatment: a population-based study. Lancet Child Adolesc Health.

[23] Wu Z, Abdullaev Z, Pratt D, Chung H-J, Skarshaug S, Zgonc V (2021). Impact of the methylation classifier and ancillary methods on CNS tumor diagnostics. Neuro Oncol.

[24] Louis DN, Perry A, Wesseling P, Brat DJ, Cree IA, Figarella-Branger D (2021). The 2021 WHO classification of tumors of the central nervous system: a summary. Neuro Oncol.

[25] Karimi S, Zuccato JA, Mamatjan Y, Mansouri S, Suppiah S, Nassiri F (2019). The central nervous system tumor methylation classifier changes neuro-oncology practice for challenging brain tumor diagnoses and directly impacts patient care. Clin Epigenetics.

[26] Capper D, Stichel D, Sahm F, Jones DTW, Schrimpf D, Sill M (2018). Practical implementation of DNA methylation and copy-number-based CNS tumor diagnostics: the Heidelberg experience. Acta Neuropathol.

[27] Jaunmuktane Z, Capper D, Jones DTW, Schrimpf D, Sill M, Dutt M (2019). Methylation array profiling of adult brain tumours: diagnostic outcomes in a large, single centre. Acta Neuropathol Commun.

[28] Bächli H, Ecker J, van Tilburg C, Sturm D, Selt F, Sahm F (2018). Molecular diagnostics in pediatric brain tumors: impact on diagnosis and clinical decision-making: a selected case series. Klin Padiatr.

[29] Prendergast SC, Strobl A-C, Cross W, Pillay N, Strauss SJ, Ye H (2020). Sarcoma and the 100,000 Genomes Project: our experience and changes to practice. J Pathol Clin Res.

[30] Miller DT, Cortés-Ciriano I, Pillay N, Hirbe AC, Snuderl M, Bui MM (2020). Genomics of MPNST (GeM) consortium: rationale and study design for multi-omic characterization of NF1-associated and sporadic MPNSTs. Genes (Basel)..

